# A Novel Positioning Technique for Olecranon Fracture Fixation

**DOI:** 10.7759/cureus.38338

**Published:** 2023-04-30

**Authors:** Sreenivasulu Metikala, Jakob J Anibas, Ryan Price, Naga Cheppalli

**Affiliations:** 1 Orthopaedics, Virginia Commonwealth University Health System, Richmond, USA; 2 Biological Sciences, University of Minnesota Twin Cities, Minneapolis, USA; 3 Orthopaedic Surgery and Rehabilitation, University of New Mexico School of Medicine, Albuquerque, USA; 4 Orthopaedics, Veterans Affairs (VA) Hospital Albuquerque, Albuquerque, USA

**Keywords:** technique, patient positioning, olecranon fracture, fracture, orthopedics

## Abstract

Various positioning techniques have been described for the osteosynthesis of olecranon fractures, each with their own pros and cons. The supine position is time-efficient and better suited in a polytrauma setting but frequently requires an assistant to maintain optimal limb positioning. Also, adequate fluoroscopic imaging is not possible without moving the operative extremity outside the sterile field. We describe a simple and reproducible method addressing these limitations while providing excellent surgical access and intraoperative imaging.

## Introduction

Olecranon fractures are common injuries accounting for 10% of all upper extremity fractures [1]. When surgical fixation is deemed necessary, optimal patient positioning is critical for ease of surgical exposure, fracture reduction, and intraoperative imaging while avoiding complications. Prone or lateral decubitus positions are often considered, but they are time-consuming, limit the ability to manage patients in a polytrauma setting, and carry an increased risk of complications such as reduced stroke volume, cardiovascular collapse, rhabdomyolysis, and nerve palsy [2-4]. In contrast, supine positioning is quick, provides easy access to the patient’s airway, allows for the management of concomitant injuries in a polytrauma setting, and facilitates gravity-assisted drainage of blood away from the operative field for better visualization. Despite the benefits of the supine position, it frequently requires a dedicated assistant to hold the elbow in the desired position throughout the surgery. Besides, to facilitate adequate biplanar fluoroscopic imaging, the operating elbow must be extended and manually held outside the sterile surgical field, posing a risk of inadvertent loss of provisional fracture reduction tools. To overcome these difficulties, we describe a novel supine positioning technique for quick and reliable access to both surgical exposure and fluoroscopy during olecranon fracture fixation.

## Technical report

Indications

This positioning technique is applicable for olecranon fractures and radial head fractures. Besides, it permits addressing any concomitant injuries to the rest of the upper extremity without repositioning.

Contraindications

The only contraindication would be concurrent thoracic trauma with major pleura, lung, or cardiac injuries. The bolster with folded arm will likely create additional pressure on the already injured chest.

Technique

**Figure 1:**
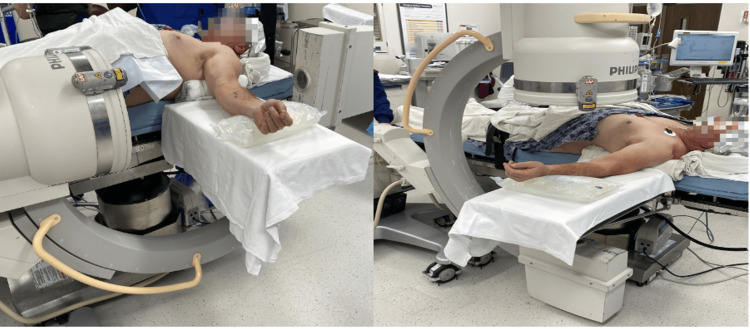
Supine positioning with rolled towel under the shoulder and 5-liter saline bag on the hand table (to be secured with tape before draping).

The patient is positioned supine with the operating extremity on a hand table. A towel roll is placed under the shoulder for elevation. A five-liter saline bag is taped to the hand table at a distance such that a radiolucent triangle (used for intramedullary tibial nailing) can later be wedged between the saline bag and the shoulder (Figure 1).

**Figure 2:**
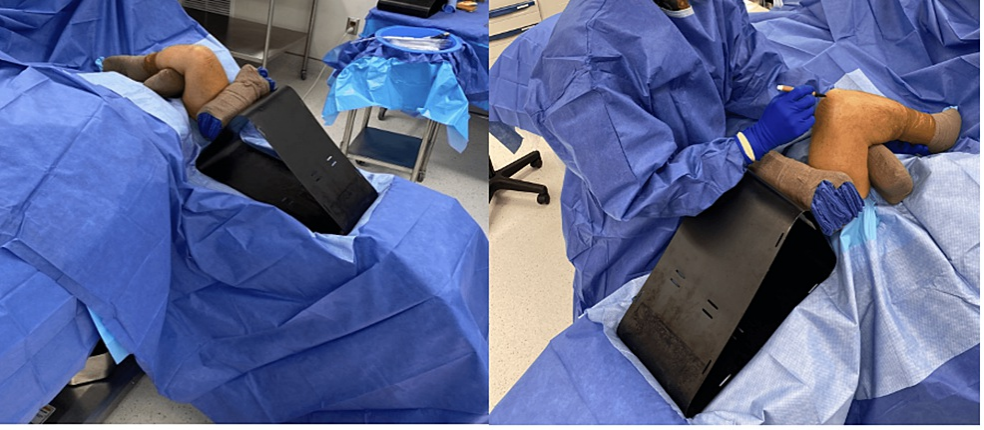
Positioning for surgical exposure. Maintenance of optimal elbow flexion (without manual assistance) using sterile bolsters and radiolucent triangle, wedged between shoulder and saline bag (concealed under sterile drapes).

The extremity is then prepped and draped in a sterile fashion. The arm is flexed over the chest wall and is supported on a sterile bolster. The radiolucent triangle is then wedged between the shoulder and saline bag with a second sterile bolster supporting the arm from sliding off the chest wall. With the arm held at optimal elbow flexion, adequate surgical exposure is now possible without needing additional manual assistance (Figure 2).

**Figure 3:**
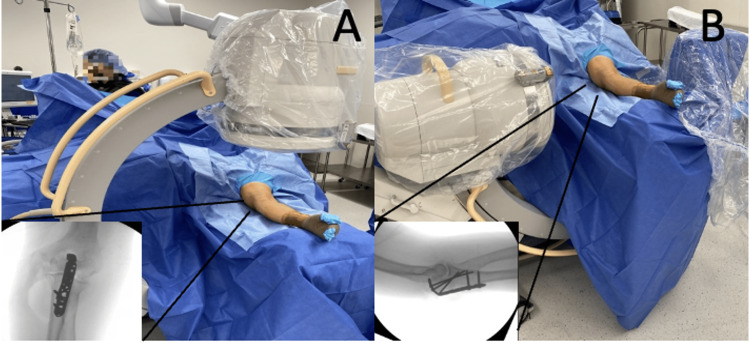
Fluoroscopic imaging. Anteroposterior (A) and lateral (B) images obtained without manipulating the operative extremity.

To obtain the standard intraoperative images, the C-arm is introduced from the foot side, parallel to the patient. Next, the elbow is extended, resting the wrist on the saline bag with overlying sterile drapes. Fluoroscopic images in multiple planes are now obtained without having to move the operative extremity (Figure 3A, 3B).

## Discussion

The described technique serves as an additional option to pre-existing supine positioning techniques with distinct advantages (Table 1).

**Table 1:** Review of supine positioning techniques for olecranon fixation

Authors	Technique	Advantages	Disadvantages
DeBernardis et al., 2020 [5]	Paint rollers & Esmarch bandage	Avoids pressure over the chest wall	Limits further flexion. Elbow needs to be extended outside the sterile field and held manually for imaging. Esmarch bandage may hinder surgical access.
Wijeratna et al., 2012 [6]	Lloyd-Davies leg support from contralateral side or L-bar from ipsilateral side	Avoids pressure over the chest wall	Limits further flexion. Elbow needs to be extended outside the sterile field and held manually for imaging.
Patel et al., 2019 [7]	Shoulder is abducted and fully externally rotated to place the elbow over a kidney dish	Quick set-up	Extreme external rotation of the shoulder is necessary for positioning. Limited access to exposure.

With the combination of sterile bolsters and a radiolucent triangle, our technique provides quick, reproducible, and seamless access to surgical exposure and fracture reduction, obviating the need for an assistant to hold the arm. The other significant advantage is that multiplanar fluoroscopic images can be obtained without moving the injured extremity, thus protecting the provisional fracture reduction. Furthermore, there is no risk of losing sterile surgical tools should any accidentally slip off the surgical field due to the underlying sterile hand table. Finally, attending to any concomitant injuries to the rest of the upper extremity is possible without repositioning.

Complications

As mentioned before, the only complication of this technique would be undesirable additional pressure over the chest wall if the patient had concurrent major chest trauma.

## Conclusions

Although other techniques have been described to allow for olecranon fracture fixation with the patient in the supine position, the positioning technique described here is advantageous given that it is quick, reproducible, and allows for easy access to the surgical site for both fracture fixation and imaging. Furthermore, our technique eliminates the need for a dedicated assistant to hold the arm throughout the procedure. With olecranon fractures being a common orthopaedic diagnosis, our technique provides the orthopaedic surgeon with a more efficient way to operate in situations where surgical fixation is necessary.
